# Case Report: Cytokine adsorption during multimodal management of hyperinflammatory toxicities following CAR-T in three patients

**DOI:** 10.3389/fimmu.2026.1968648

**Published:** 2026-10-29

**Authors:** Haoran Xu, Fangce Wang, Yuqiu Lu, Wenjun Zhang, Yingying Zhang, Aibin Liang

**Affiliations:** 1 Department of Hematology, Shanghai Tongji Hospital, Tongji University School of Medicine, Shanghai, China; 2 Clinical Research Ward of Cancer Center, Shanghai Tongji Hospital, Tongji University School of Medicine, Shanghai, China; 3 Department of Nephrology, Shanghai Tongji Hospital, Tongji University School of Medicine, Shanghai, China

**Keywords:** car-t, case report, cytokine adsorption, cytokine release syndrome, hyperinflammatory states

## Abstract

Chimeric antigen receptor T cell (CAR-T) has advanced rapidly in hematologic malignancies and even solid tumors, but post-treatment adverse reactions remain a major risk. Most adverse reactions are associated with inflammatory responses and cytokine release, such as cytokine release syndrome. Cytokine adsorption is an extracorporeal blood-purification approach designed to adsorb circulating inflammatory mediators. We report three patients with CAR-T-associated CRS, CRS with ICANS, or CRS with IEC-HS who received CA330-based cytokine adsorption as part of multimodal management. Clinical recovery and reductions in selected inflammatory markers were observed during the treatment course, and no procedure-related adverse events were documented. However, because adsorption overlapped with anti-IL-6 therapy, corticosteroids, anti-infective and supportive care, and the natural evolution of these toxicities, its independent contribution could not be determined. These observations support the feasibility of this approach and warrant prospective evaluation.

## 1 Introduction

Chimeric antigen receptor T cell (CAR-T) therapy has made significant progress in the treatment of hematologic malignancies, with multiple commercial products approved for hematologic malignancies (1, 2). With the wide application of CAR-T, multicenter observational studies have revealed a high incidence of hematologic toxicity and severe infectious complications following CAR-T treatment, and these post-treatment complications have also limited the further clinical application of this immunotherapeutic approach (3). The major acute adverse effects of CAR-T therapy include cytokine release syndrome (CRS), immune effector cell-associated neurotoxicity syndrome (ICANS), immune effector cell-associated hemophagocytic lymphohistiocytosis-like syndrome (IEC-HS), infections, and others (4–7). Current management includes supportive care, IL-6-pathway blockade for clinically significant CRS, and corticosteroids for severe or refractory CRS and ICANS. However, rapidly progressive or overlapping inflammatory syndromes, particularly when accompanied by infection or organ dysfunction, remain difficult to diagnose and manage. This clinical challenge has prompted interest in extracorporeal cytokine adsorption as a potential adjunctive treatment (8, 9).

Cytokine Adsorption is an *in vitro* blood purification technique that removes excessively released pro-inflammatory and anti-inflammatory cytokines from the blood through specialized adsorption columns integrated into an extracorporeal circulation device. The primary clinical application purpose is to remove substances such as autoantibodies, immune complexes, and cytokines from the patient’s plasma (10–12). Cytokine Adsorption has been reported as an adjunct in isolated cases of severe CRS or ICANS, but evidence is limited to uncontrolled reports and its independent clinical effect remains uncertain (13–15).

## 2 Case description

Between September 2025 and February 2026, 50 patients received CAR-T cell therapy at our center. Eight patients met the criteria for extracorporeal cytokine adsorption: grade ≥3 CRS, CRS with ICANS, or CRS with IEC-HS; CRS and ICANS were graded according to the 2019 ASTCT consensus criteria, whereas IEC-HS was diagnosed and graded according to the criteria proposed by Hines et al (5, 16). Of these, three patients and their legal representatives provided written informed consent and underwent cytokine adsorption; five declined. No other patient received cytokine adsorption during this period. The three included patients comprised one case of grade 3 CRS, one of CRS with ICANS, and one of CRS with IEC-HS. We described the clinical grading, key inflammatory markers, and cytokine trajectories before and after cytokine adsorption. Serial serum creatinine (sCr) and estimated glomerular filtration rate (eGFR) were reviewed throughout the clinical course. Hourly urine output was not recorded with sufficient completeness for KDIGO assessment; therefore, AKI was evaluated using the serum creatinine component of the KDIGO criteria only.

Vascular access was established using a temporary, non-tunneled, non-cuffed central venous dialysis catheter. Because the CA330 is a stand-alone hemoadsorption cartridge, a continuous venovenous hemofiltration (CVVH) platform and tubing set were used to establish and monitor the extracorporeal circuit. The CA330 cytokine adsorption column (product model CA330; Jafron Biomedical Co., Ltd., Zhuhai, China) was connected in series downstream of the hemofilter (17, 18). The hemofilter was an Aquamax HF12 (Nikkiso Belgium bv, Tienen, Belgium), comprising a high-flux polyethersulfone hollow-fiber membrane with an effective surface area of 1.20 m², a membrane thickness of 30 μm, and a fiber inner diameter of 200 μm. The extracorporeal circuit was operated only during each 3-hour hemofiltration–hemoperfusion session and was discontinued between sessions.Therefore, continuous 24-hour hemofiltration was not performed. Blood flow was maintained at 200 mL/min, and replacement fluid was delivered at 2,000 mL/h. Net ultrafiltration was prescribed at 0 mL.

Regional citrate anticoagulation was administered at 240 mL/h according to the institutional protocol, with target post-filter and systemic ionized calcium concentrations of 0.25–0.35 mmol/L and 1.00–1.25 mmol/L, respectively. No visible filter clotting occurred during treatment (clotting grade 0). During the procedure, IL-6, ferritin, norepinephrine dose, and blood pressure changes were monitored. All three patients completed three adsorption sessions as part of multimodal treatment; their subsequent clinical and laboratory courses are described below.

### 2.1 Case 1

A 68-year-old female patient was diagnosed with ALK-negative anaplastic large cell lymphoma 10 years ago and achieved complete remission (CR) after several cycles of chemotherapy. Two years ago, the patient developed sudden confusion accompanied by high fever. The cerebrospinal fluid (CSF) test indicated a recurrence and metastasis of lymphoma in the central nervous system. She was treated with intrathecal chemotherapy consisting of cytarabine, methotrexate, and dexamethasone, along with systemic chemotherapy comprising brentuximab vedotin, gemcitabine, cisplatin, and dexamethasone. PET-CT assessment indicated a poor treatment response and multiple new lesions and a subsequent biopsy suggested transformation to diffuse large B-cell lymphoma. After admission, the patient received lymphodepletion with fludarabine and cyclophosphamide, and then CD19 CAR-T was infused. On day (D) 0 after infusion, there was a peak of high fever at night reaching 38.8°C, and blood inflammatory cytokines and ferritin were significantly elevated. Considering it as grade 1 CRS, and supportive treatment including anti-infection and hepatoprotective therapy was administered. Subsequently, on D2, the patient developed the highest recorded temperature of 40.0°C, with CRS reaching a maximum of grade 2. Thereafter, the patient developed somnolence and lethargy, and the immune effector cell-associated encephalopathy (ICE) score decreased to the lowest 5 points on D5. A lumbar puncture was performed, suggesting a significant increase in inflammatory factors in the CSF, and these findings were considered consistent with grade 2 ICANS (16). No obvious symptoms of epilepsy or increased intracranial pressure were observed.

The patient received siltuximab 400 mg on D2, D3, D4, and D6, and underwent cytokine adsorption on D4, D5, and D6. At the same time, dexamethasone was administered intravenously at 10 mg on D4, 5 mg on D5, 10 mg on D6, 15 mg on D7, 10 mg daily from D8 to D11, and 5 mg on D12, and 40 mg of methylprednisolone was used starting from D13. Then, dexamethasone was given at 2.5 mg daily from day 14 to day 18 (**Figure 1a**).

**Figure 1:**
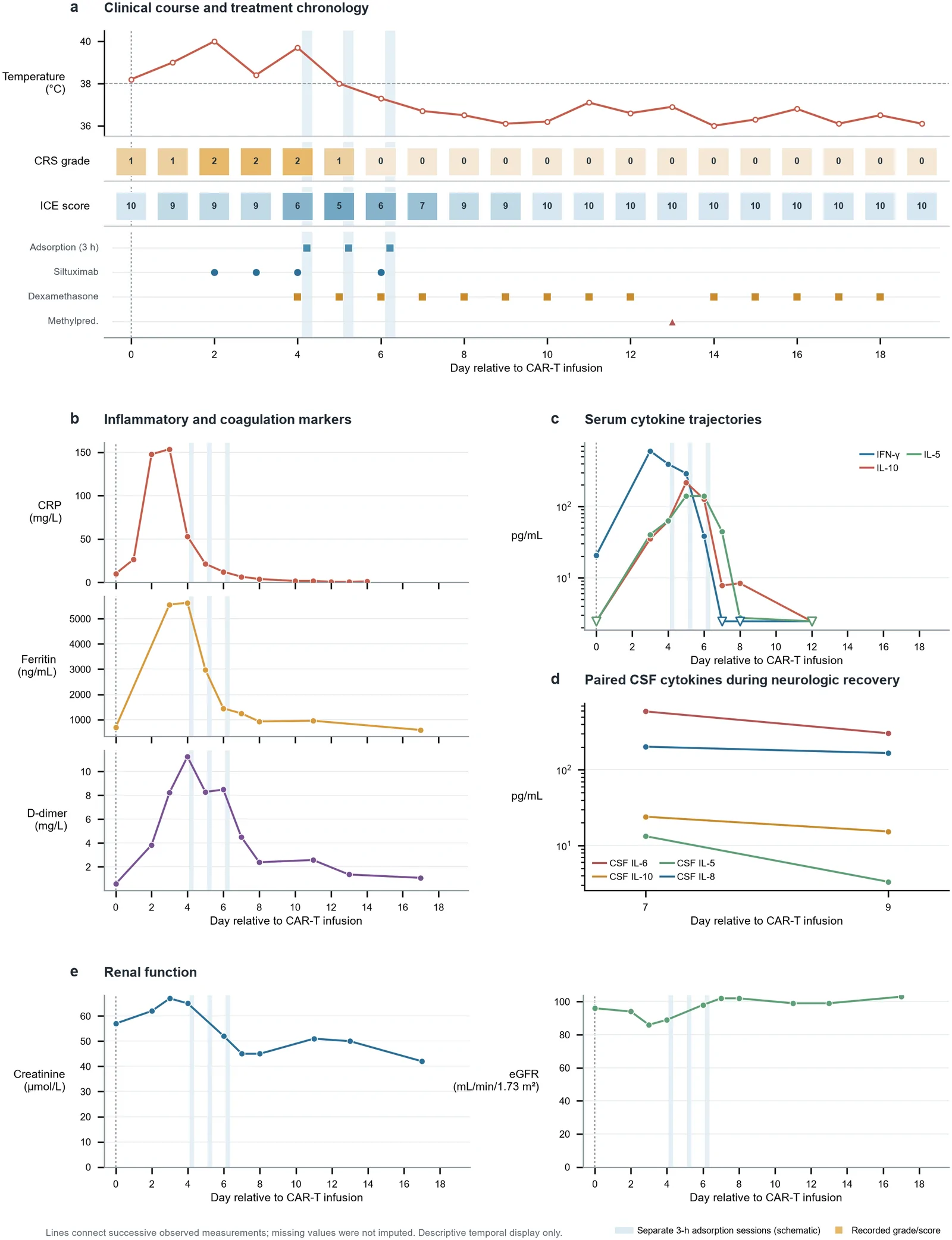
Clinical course and selected biomarker trajectories in case 1. D0 denotes CAR-T infusion. **(a)** Temperature, CRS grade, ICE score, three separate 3-hour adsorption sessions on D4–D6, and concomitant siltuximab and corticosteroids. **(b)** CRP, ferritin, and D-dimer. **(c)** Serum IFN-γ, IL-10, and IL-5. **(d)** Paired CSF cytokines on D7 and D9. **(e)** Creatinine and eGFR; creatinine-based KDIGO criteria for AKI were not met. Blood samples were collected at approximately 7:00 AM before same-day treatment. The three narrow light-blue vertical boxes indicate the separate 3-hour adsorption sessions and are positioned schematically after the corresponding pre-treatment sampling points; box widths are not to scale. Lines connect observed measurements; missing values were not imputed.

During multimodal treatment, the CRS level returned to grade 0 on day 6; the ICE score was 7 on D7 and 9 on D9, and recovered to 10 on D10. The patient’s neurological symptoms improved significantly, and body temperature dropped to below 38°C on D5 (**Figure 1a**). In terms of laboratory findings, C-reactive protein (CRP) peaked at 153.6 mg/L on D3 and decreased to 12.3 mg/L on D6, a reduction of 92.0%; ferritin peaked at 5632 ng/mL on D4 and decreased to 1446 ng/mL on D6, a reduction of 74.3%; and D-dimer peaked at 11.25 mg/L on D4 (**Figure 1b**). Serum levels of IFN-γ, IL-10 and IL-5 decreased overall (**Figure 1c**). CSF analysis showed that IL-6 decreased from 590.22 to 303.99 pg/mL, IL-10 from 24.06 to 15.37 pg/mL, IL-5 from 13.32 to 3.33 pg/mL, and IL-8 from 201.90 to 166.48 pg/mL (**Figure 1d**). Serum creatinine remained between 42 and 67 μmol/L, with an eGFR of 86–103 mL/min/1.73 m². Creatinine-based KDIGO criteria for AKI were not met. The patient was discharged after symptom improvement and received oral zanubrutinib as maintenance therapy (**Figure 1e**). At the 6-month follow-up, PET-CT evaluation indicated a partial response.

### 2.2 Case 2

A 57-year-old female patient presented with unsteady gait and declining calculation ability one year ago and was referred to another hospital for further examination. PET-CT and biopsy suggested primary central nervous system diffuse large B-cell lymphoma. Subsequently, she was treated with rituximab 600 mg, methotrexate 10 g, and dexamethasone 15 mg, followed by a regimen of rituximab, cytarabine, and orelabrutinib. However, the treatment response was poor, and the disease progressed. After admission, the patient received lymphodepletion with fludarabine 147 mg and cyclophosphamide 1470 mg, followed by infusion of CD19 CAR-T cell therapy.On D1 after CAR-T infusion, the patient developed a fever of 39.4°C. Chest radiography showed findings suggestive of pulmonary infection, and the procalcitonin concentration was 1.65 ng/mL. Empirical antimicrobial treatment with cefepime was initiated at 2.0 g intravenously twice daily. The patient subsequently developed persistent fever, generalized edema, and hypotension (blood pressure, 84/46 mmHg), requiring vasoactive support with dopamine at 3.3 μg/kg/min for 24 h. The serum lactate concentration was 2.85 mmol/L, and the SOFA score was 6. Based on the temporal relationship to CAR-T infusion and the accompanying hemodynamic deterioration, grade 3 CRS was considered; however, concomitant pulmonary infection and septic shock were also diagnosed. Because CRS and sepsis occurred concurrently and shared fever, hypotension, and organ dysfunction as clinical manifestations, their respective contributions to the hemodynamic deterioration could not be distinguished with certainty.

The patient received four doses of siltuximab 400 mg on D1, D2, D4, and D6, and underwent three sessions of cytokine adsorption from D5 to D7. The patient’s CRS reached grade 3 on D3. During the treatment course, the body temperature decreased to below 38°C on D6. On D8, the patient’s overall condition improved, oxygen saturation increased to 99%, and both CRS and sepsis were considered to have improved (**Figure 2a**). CRP decreased from 146.8 mg/L on D2 to 8.1 mg/L on D7, a reduction of 94.5%; ferritin decreased from a peak of 766 ng/mL on D3 to 525 ng/mL on D7, a reduction of 31.5%. D-dimer peaked at 13.5 mg/L FEU on D5, and subsequently was 1.57, 1.76, 1.35, and 1.26 mg/L FEU on D9, D11, D14, and D17, respectively (**Figure 2b**). Although CRS grade and selected inflammatory markers had already begun to improve before the first adsorption session, IFN-γ, IL-5, and IL-10 decreased rapidly around the period of cytokine adsorption(**Figure 2c**), while the recovery of IL-6 and IL-2Rα was slightly delayed compared to the clinical remission of CRS (**Figure 2d**). Serum creatinine ranged from 47 to 82 μmol/L, with an eGFR of 72–107 mL/min/1.73 m²(**Figure 2e**). Creatinine-based KDIGO criteria for AKI were not met. The patient was discharged after symptom relief and received sintilimab as maintenance therapy. At the 6-month follow-up, PET-CT evaluation indicated that the disease had achieved CR.

**Figure 2:**
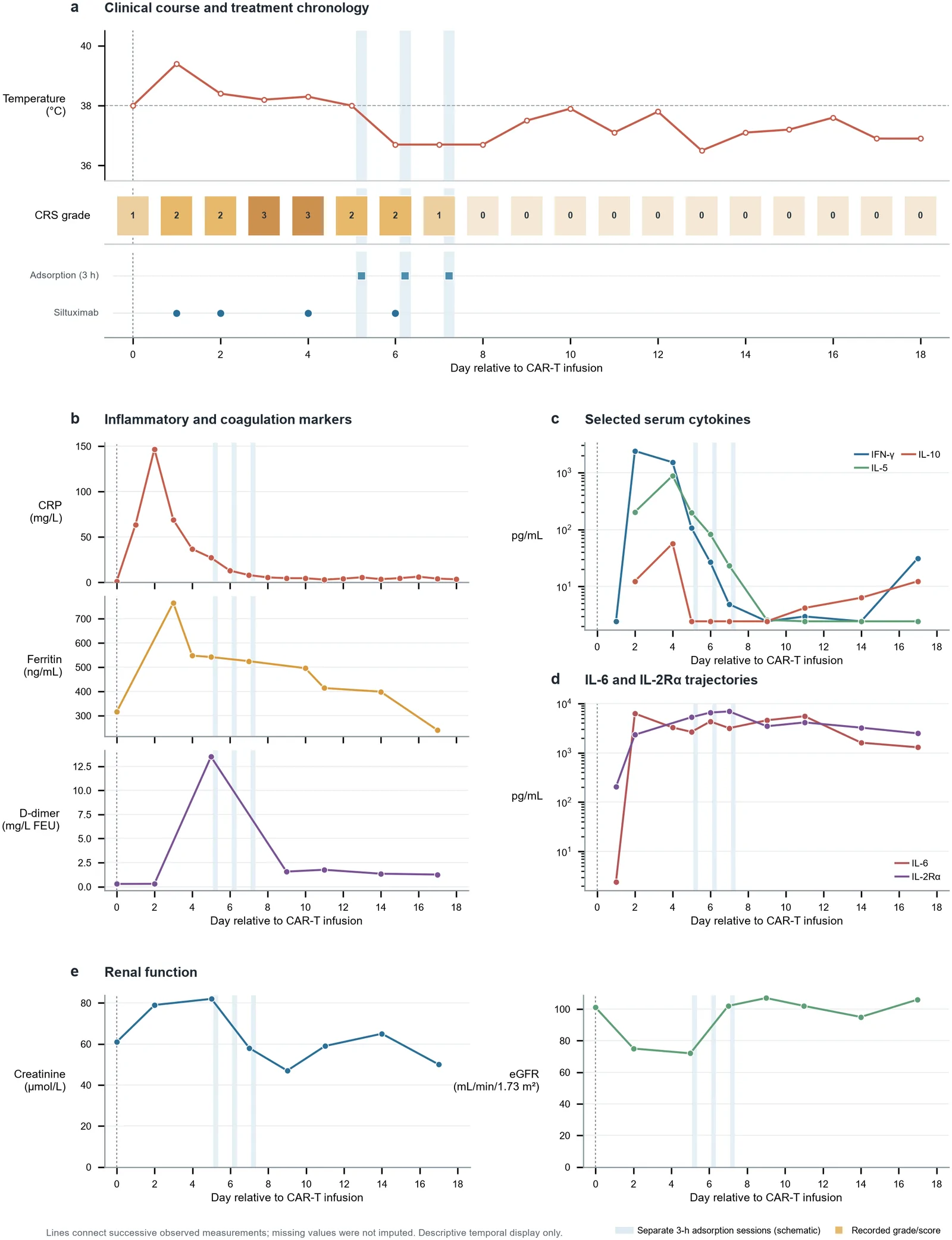
Clinical course and selected biomarker trajectories in case 2. D0 denotes CAR-T infusion. **(a)** Temperature, CRS grade, three separate 3-hour adsorption sessions on D5–D7, and concomitant siltuximab. **(b)** CRP, ferritin, and D-dimer. **(c)** Serum IFN-γ, IL-5, and IL-10. **(d)** IL-6 and IL-2Rα, which remained elevated after CRS improvement. **(e)** Creatinine and eGFR; creatinine-based KDIGO criteria for AKI were not met. Blood samples were collected at approximately 7:00 AM before same-day treatment. The three narrow light-blue vertical boxes indicate the separate 3-hour adsorption sessions and are positioned schematically after the corresponding pre-treatment sampling points; box widths are not to scale. Lines connect observed measurements; missing values were not imputed.

### 2.3 Case 3

A 67-year-old male was diagnosed with multiple myeloma (MM) two years ago. Fluorescence *in situ* hybridization revealed IGH/MYC gene fusion, 1q amplification, and deletion of chromosome 17 in 10% of nuclei, defining the disease as “triple-hit” MM. At the same time, PET/CT revealed liver and kidney involvement, suggesting extramedullary infiltration lesions, consistent with high-risk MM. Due to pancytopenia, the patient was unable to tolerate chemotherapy with bortezomib, lenalidomide, and dexamethasone. With further progression of extramedullary disease, the patient received BCMA-directed CAR-T cell infusion (equecabtagene autoleucel) one year ago and developed mild CRS after the infusion. Three months after the treatment, PET-CT and biopsy indicated relapse of MM. Then, the patient received radiotherapy at a local hospital with a total dose of 20 Gy delivered in 5 fractions. However, the treatment response was poor. For the current admission, the patient received lymphodepletion with cyclophosphamide 1854 mg and fludarabine 184.5 mg. Two days later, BCMA/GPRC5D CAR-T cells were infused. After infusion, the patient developed persistent fever and the blood test showed a progressive increase in IL-6. It was considered as CRS reaction. After treatment with acetaminophen and indomethacin suppository, the effect was not satisfactory. On D4, his temperature reached 39.5 °C, accompanied by lethargy, poor mental status, and pulmonary edema. Grade 2 CRS was considered. On D7, the patient developed a rapid increase in ferritin and persistently elevated IL-2Rα levels, accompanied by thrombocytopenia, elevated D-dimer, and a decrease in fibrinogen to 0.8 g/L, consistent with grade 2 IEC-HS (5).

The patient received tocilizumab 320 mg on D4, siltuximab 400 mg on D5 and D6, and subsequently underwent three sessions of cytokine adsorption from D7 to D9 (**Figure 3a**). The patient’s CRP reached 173.7 mg/L on D4. After treatment, it dropped to 26.3 mg/L on D9, a decrease of 84.9%. Ferritin increased gradually from 825 ng/mL on D2, reached 4316 ng/mL on D8, decreased to 1754 ng/mL on D9 after cytokine adsorption, and further declined to 832 ng/mL on D10 (**Figure 3b**). IL-2Rα exceeded the upper limit of detection of 7500 pg/mL from D6 to D8, and decreased to 5694, 2122, and 650 pg/mL on D9, D10, and D11, respectively (**Figure 3c**). Along with the patient’s body temperature dropping, it was considered that IEC-HS and CRS had been relieved. Additionally, the platelet count decreased from 58 × 10^9^/L on D0 to 20 × 10^9^/L on D5. D-dimer increased from 0.50 mg/L FEU on D2 to 15.02 mg/L FEU on D8, after which it began to decline gradually but had not yet returned to normal levels (**Figure 3d**). Serum creatinine increased from 102 μmol/L on D0 to 237 μmol/L on D5 and 423 μmol/L on D7, accompanied by a decrease in eGFR from 69 to 25 and 13 mL/min/1.73 m², respectively. This was consistent with KDIGO stage 3 AKI based on serum creatinine criteria. Serum creatinine subsequently decreased to 84 μmol/L on D9 and 71 μmol/L on D10 (**Figure 3e**). The patient’s symptoms subsequently improved, and the patient was discharged in stable condition.

**Figure 3:**
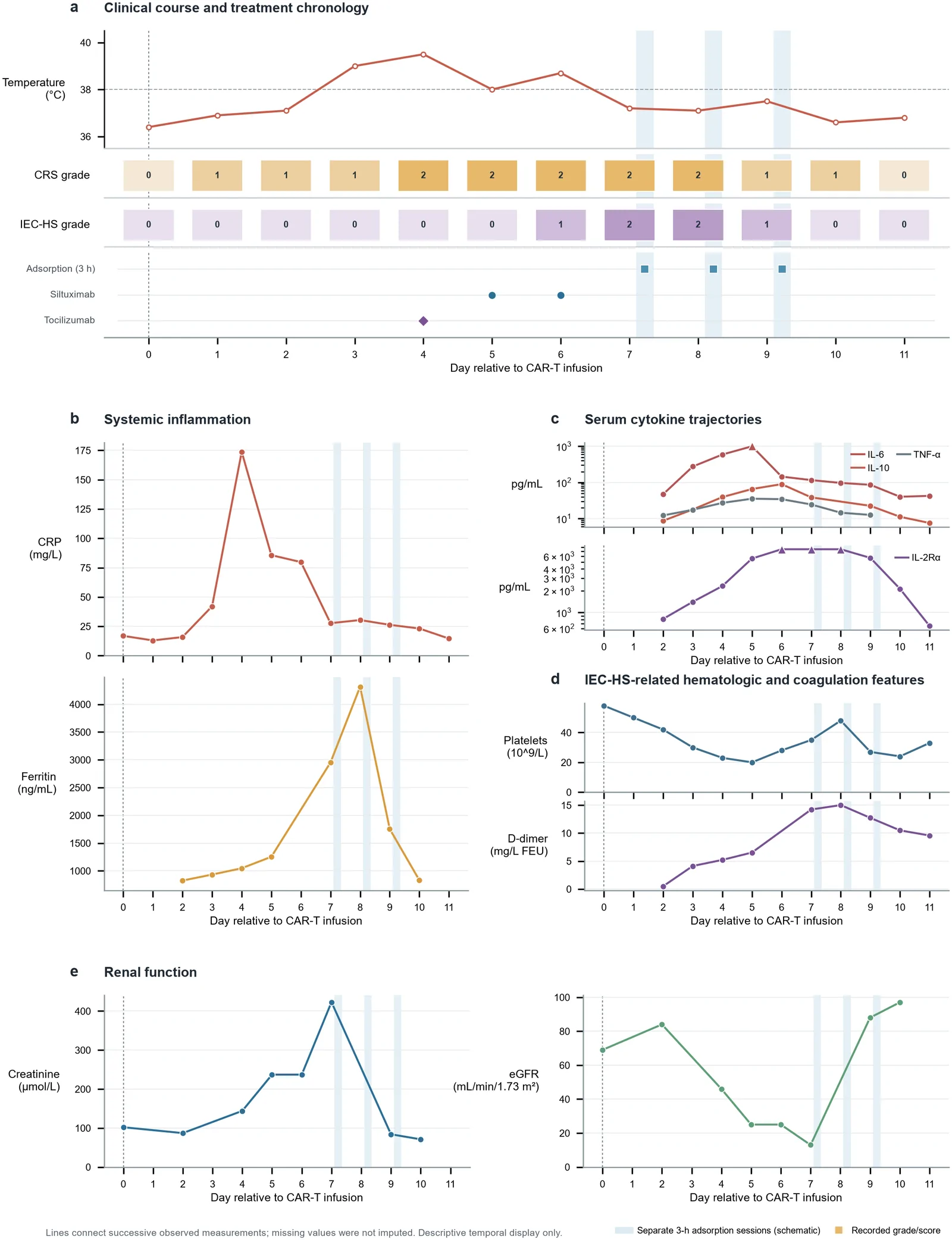
Clinical course and selected biomarker trajectories in case 3. D0 denotes CAR-T infusion. **(a)** Temperature, CRS and IEC-HS grades, three separate 3-hour adsorption sessions on D7–D9, and concomitant siltuximab and tocilizumab. **(b)** CRP and ferritin. **(c)** Serum IL-6, IL-10, TNF-α, and IL-2Rα. **(d)** Platelet count and D-dimer. **(e)** Creatinine and eGFR; creatinine increased from 102 μmol/L on D0 to 423 μmol/L on D7, meeting serum-creatinine criteria for KDIGO stage 3 AKI, and decreased to 84 μmol/L on D9. Blood samples were collected at approximately 7:00 AM before same-day treatment; therefore, the D7 renal measurements preceded the first adsorption session. The three narrow light-blue vertical boxes indicate the separate 3-hour adsorption sessions and are positioned schematically after the corresponding pre-treatment sampling points; box widths are not to scale. Lines connect observed measurements; missing values were not imputed.

## 3 Discussion

In this three-patient case series, CA330-based cytokine adsorption was feasible to deliver as part of multimodal management of different CAR-T-associated hyperinflammatory toxicities. Clinical improvement and decreases in selected inflammatory markers occurred during the overall treatment course. However, adsorption overlapped with anti-IL-6 therapy, corticosteroids, anti-infective and supportive care, and the natural evolution of the toxicities. Moreover, several clinical or laboratory variables were already improving before adsorption was initiated in Cases 2 and 3. Therefore, the independent contribution of cytokine adsorption cannot be determined from these uncontrolled observations. AKI is a clinically relevant complication after CAR-T-cell therapy and has been associated with severe CRS and adverse outcomes. In the present series, Cases 1 and 2 did not meet creatinine-based KDIGO criteria for AKI, whereas Case 3 developed stage 3 AKI followed by rapid renal recovery. Because urine output was unavailable and renal recovery occurred during multimodal treatment, the contribution of cytokine adsorption to renal recovery cannot be determined (19).

From the perspective of pathophysiological mechanisms, the excessive release of pro-inflammatory cytokines following CAR-T therapy is a central driver of CRS, ICANS, and IEC-HS. These factors not only activate the systemic inflammatory cascade reaction but also can damage the blood-brain barrier, induce microvascular dysfunction, and cause coagulation abnormalities (20, 21). Cytokine adsorption is designed to adsorb mediators across a broad molecular-weight range; whether this translates into improved organ outcomes in CAR-T-associated toxicities remains uncertain (22–24). Most previously reported applications of extracorporeal cytokine removal for CAR-T-related toxicities used the CytoSorb cartridge, whereas CA330 was used in the present series. Both are nonselective polymer-resin adsorbers but differ in material properties, treatment protocols, and evidence base. CA330 has a nominal 5–60-kDa adsorption range and was applied for 3 hours per session; CytoSorb is generally used for longer periods. Evidence for CA330 in CAR-T-associated toxicities remains limited to *in vitro* and sepsis-related studies (17, 18).

Free IL-6, with a molecular weight of approximately 21–26 kDa, lies within the nominal adsorption range and may therefore be removed by CA330. In contrast, siltuximab, an anti-IL-6 monoclonal antibody, and tocilizumab, an anti-IL-6 receptor monoclonal antibody, both have molecular weights of approximately 148 kDa. Substantial adsorption of the intact antibodies is therefore unlikely on the basis of molecular size (25). However, no CA330-specific pharmacokinetic study has directly measured their extraction, and clinically relevant removal cannot be completely excluded. Moreover, IL-6 concentrations after siltuximab or tocilizumab should be interpreted cautiously because these drugs may alter circulating IL-6 kinetics or interfere with some cytokine assays (26). Resin hemoadsorption may nonselectively remove concomitant medications, including anti-infective agents, although the extent of removal is drug- and device-specific. Cefepime was therefore administered after each adsorption session. Because drug concentrations were not measured, the effect of CA330 on cefepime exposure remains unknown (27, 28).

The optimal timing and number of adsorption sessions remain undefined. The three patients differed substantially in toxicity phenotype, disease severity, concomitant treatments, and the trajectories already present before adsorption. Therefore, the apparent differences in biomarker recovery cannot be attributed to earlier or later initiation of adsorption. These temporal patterns should be considered hypothesis-generating rather than comparative evidence of treatment timing (29). In Case 2, clinical improvement and CRP reduction preceded the normalization of IL-6 and IL-2Rα. Blood samples were collected at approximately 7:00 AM before same-day treatment, without immediate paired pre- and post-adsorption sampling. Therefore, these cytokine trajectories cannot establish direct cytokine removal by CA330 and may also reflect ongoing production, redistribution, natural disease evolution, and concomitant treatment (26).

In terms of safety, none of the patients in this cohort experienced complications directly related to the adsorption procedure, such as hypotension, bleeding or thrombosis. Although two patients had thrombocytopenia and coagulopathy, the treatment was completed successfully under regional citrate anticoagulation and close monitoring. This suggests that cytokine adsorption is feasible in patients with coagulopathy following CAR-T therapy. However, caution should still be exercised regarding electrolyte imbalances and coagulation risks, especially for those with impaired liver and kidney function (30, 31).

In summary, these cases document the technical feasibility of short duration CA330 based cytokine adsorption as an adjunct to multimodal management. They do not establish treatment efficacy, optimal initiation timing, or the appropriate number of sessions. Prospective studies with prespecified eligibility criteria, standardized concomitant therapy, and paired pre- and post-session sampling are required.

## 4 Patient perspective

When the three patients recalled the high fever, confusion of consciousness and extreme fatigue after CAR-T treatment, they all stated that it was the most difficult stage of the entire treatment, and they were once worried that they might lose the opportunity for relief. After receiving the treatment combining cytokine adsorption with medication, they generally experienced a drop in body temperature and a significant improvement in mental state within 1 to 2 days. Their blood pressure also stabilized. One of the patients particularly mentioned, “Being able to clearly recognize family members again was a turning point that made me believe I would recover”.

## Data Availability

The de-identified data supporting the conclusions of this article will be made available by the corresponding author upon reasonable request, subject to applicable ethical and privacy restrictions.

## References

[1] Iacoboni G Rejeski K Villacampa G van Doesum JA Chiappella A Bonifazi F . Real-world evidence of brexucabtagene autoleucel for the treatment of relapsed or refractory mantle cell lymphoma. Blood Adv. (2022) 6:3606–10. doi: 10.1182/bloodadvances.2021006922 35271707 PMC9631549

[2] Kamdar M Bartlett NL . From breakthroughs to blueprints: evolving evidence and future directions in relapsed and refractory large b-cell lymphoma. Bloodblood. (2026) 147:2879–94. doi: 10.1182/blood.2025030859 41871044

[3] Rejeski K Wang Y Albanyan O Munoz J Sesques P Iacoboni G . Thecar‐hematotox score identifies patients at high risk for hematological toxicity, infectious complications, and poor treatment outcomes following brexucabtagene autoleucel for relapsed or refractorymcl. Am J Hematol. (2023) 98:1699–710. doi: 10.1002/ajh.27056 37584447 PMC10659121

[4] Morris EC Neelapu SS Giavridis T Sadelain M . Cytokine release syndrome and associated neurotoxicity in cancer immunotherapy. Nat Rev Immunol. (2022) 22:85–96. doi: 10.1038/s41577-021-00547-6 34002066 PMC8127450

[5] Hines MR Knight TE McNerney KO Leick MB Jain T Ahmed S . Immune effector cell-associated hemophagocytic lymphohistiocytosis-like syndrome. Transplant Cell Ther. (2023) 29:431–8. doi: 10.1016/j.jtct.2023.03.006 36906275 PMC10330221

[6] Logue JM Zucchetti E Bachmeier CA Krivenko GS Larson V Ninh D . Immune reconstitution and associated infections following axicabtagene ciloleucel in relapsed or refractory large b-cell lymphoma. Haematologica (Roma). (2021) 106:978–86. doi: 10.3324/haematol.2019.238634 32327504 PMC8017820

[7] Ke M Kang L Wang L Yang S Wang Y Liu H . Car-t therapy alters synthesis of platelet-activating factor in multiple myeloma patients. J Hematol Oncol. (2021) 14:90. doi: 10.1186/s13045-021-01101-6 34108020 PMC8191024

[8] Santomasso BD Nastoupil LJ Adkins S Lacchetti C Schneider BJ Anadkat M . Management of immune-related adverse events in patients treated with chimeric antigen receptor t-cell therapy: asco guideline. J Clin Oncol. (2021) 39:3978–92. doi: 10.1200/JCO.21.01992 34724386

[9] Maus MV Alexander S Bishop MR Brudno JN Callahan C Davila ML . Society for immunotherapy of cancer (sitc) clinical practice guideline on immune effector cell-related adverse events. J Immunother Cancer. (2020) 8:e001511. doi: 10.1136/jitc-2020-001511 33335028 PMC7745688

[10] Saldaña-Gastulo JJC Llamas-Barbarán MDR Coronel-Chucos LG Hurtado-Roca Y . Cytokine hemoadsorption with cytosorb® in patients with sepsis: a systematic review and meta-analysis. Crit Care Sci. (2023) 35:217–25. doi: 10.5935/2965-2774.20230289-en 37712812 PMC10406402

[11] Mitzner S Kogelmann K Ince C Molnár Z Ferrer R Nierhaus A . Adjunctive hemoadsorption therapy with cytosorb in patients with septic/vasoplegic shock: a best practice consensus statement. J Clin Med. (2023) 12:7199. doi: 10.3390/jcm12237199 38068250 PMC10707447

[12] Jansen A Waalders NJB van Lier DPT Kox M Pickkers P . Cytosorb hemoperfusion markedly attenuates circulating cytokine concentrations during systemic inflammation in humans *in vivo*. Crit Care. (2023) 27:117. doi: 10.1186/s13054-023-04391-z 36945034 PMC10029173

[13] Buhlmann A Rom E Schweiger G Schneidawind D David S . Extracorporeal cytokine adsorption as therapeutic option for immune effector cell-associated neurotoxicity syndrome. Neurol Sci. (2025) 46:479–81. doi: 10.1007/s10072-024-07812-1 39417926 PMC11698748

[14] Cavallaro G Ferrari S Pollastri A Di Credico E Meli C Rondi M . Extracorporeal cytokine hemadsorption for the treatment of severe steroid refractory cytokine release syndrome secondary to t-cell immunotherapy for b-cell malignancies: a case series. Hematol Oncol. (2026) 44:e70210. doi: 10.1002/hon.70210 42329890 PMC13286135

[15] Singbartl K Rosenthal A Leis J Patel B Sen A . Novel use of extracorporeal blood purification for treatment of severe, refractory neurotoxicity after chimeric antigen receptor t-cell therapy—a case report. Crit Care Explor. (2021) 3:e472. doi: 10.1097/CCE.0000000000000472 34235458 PMC8245117

[16] Lee DW Santomasso BD Locke FL Ghobadi A Turtle CJ Brudno JN . Astct consensus grading for cytokine release syndrome and neurologic toxicity associated with immune effector cells. Biol Blood Marrow Transplant. (2019) 25:625–38. doi: 10.1016/j.bbmt.2018.12.758 30592986 PMC12180426

[17] Reis T Premužić V Kitawara K Noronha IL Ronco C . Basic mechanisms of hemoadsorption: incumbency for better clinical utility. Blood Purif. (2026) 55:340–53. doi: 10.1159/000548120 40875731

[18] Lorenzin A Perin N De Cal M Zanella M Ronco C . Endotoxin and cytokine removal with a new CA sorbent cartridge. Blood Purif. (2026) 55(6-7):464–72. doi: 10.1159/000547419 40659007

[19] Russo E Gambella M Raiola AM Beltrametti E Zanetti V Chirco G . Acute kidney injury in hematological patients treated with car-t cells: risk factors, clinical presentation and impact on outcomes. Sci Rep. (2024) 14:26886. doi: 10.1038/s41598-024-77720-z 39506012 PMC11542077

[20] Norelli M Camisa B Barbiera G Falcone L Purevdorj A Genua M . Monocyte-derived il-1 and il-6 are differentially required for cytokine-release syndrome and neurotoxicity due to car t cells. Nat Med. (2018) 24:739–48. doi: 10.1038/s41591-018-0036-4 29808007

[21] Giavridis T van der Stegen SJC Eyquem J Hamieh M Piersigilli A Sadelain M . Car t cell-induced cytokine release syndrome is mediated by macrophages and abated by il-1 blockade. Nat Med. (2018) 24:731–8. doi: 10.1038/s41591-018-0041-7 29808005 PMC6410714

[22] Ankawi G Fan W Pomarè Montin D Lorenzin A Neri M Caprara C . A new series of sorbent devices for multiple clinical purposes: current evidence and future directions. Blood Purif. (2018) 47:94–100. doi: 10.1159/000493523 30253409

[23] Köhler T Schwier E Praxenthaler J Kirchner C Henzler D Eickmeyer C . Therapeutic modulation of the host defense by hemoadsorption with cytosorb®—basics, indications and perspectives—a scoping review. Int J Mol Sci. (2021) 22:12786. doi: 10.3390/ijms222312786 PMC8657779 34884590

[24] Bottari G Merli P Guzzo I Stoppa F Ruggeri A Di Nardo M . Multimodal therapeutic approach of cytokine release syndrome developing in a child given chimeric antigen receptor-modified t cell infusion. Crit Care Explor. (2020) 2:e71. doi: 10.1097/CCE.0000000000000071 32166291 PMC7063902

[25] Scheier J Nelson PJ Schneider A Colombier S Kindgen-Milles D Deliargyris EN . Mechanistic considerations and pharmacokinetic implications on concomitant drug administration during cytosorb therapy. Crit Care Explor. (2022) 4:e688. doi: 10.1097/CCE.0000000000000688 35783552 PMC9243242

[26] Chen F Teachey DT Pequignot E Frey N Porter D Maude SL . Measuring il-6 and sil-6r in serum from patients treated with tocilizumab and/or siltuximab following car t cell therapy. J Immunol Methods. (2016) 434:1–8. doi: 10.1016/j.jim.2016.03.005 27049586 PMC5490247

[27] Schneider AG André P Scheier J Schmidt M Ziervogel H Buclin T . Pharmacokinetics of anti-infective agents during cytosorb hemoadsorption. Sci Rep. (2021) 11:10493. doi: 10.1038/s41598-021-89965-z 34006946 PMC8131695

[28] Paul R Ansari AS Ballesteros D Klaus T Honoré PM . Current knowledge on the impact of extracorporeal blood purification devices on drug clearance - systematic search & Narrative review. Blood Purif. (2025) 54:1–22. doi: 10.1159/000547477 40875683 PMC12503691

[29] Esposito P Gambella M Russo E Raiola AM Beltrametti E Conti N . Hemoadsorption as a supportive strategy for severe toxicity associated with chimeric antigen receptor t-cell therapy: a case series. Kidney Med. (2025) 7:101001. doi: 10.1016/j.xkme.2025.101001 40510619 PMC12152617

[30] Jacobs R Verbrugghe W Dams K Roelant E Couttenye MM Devroey D . Regional citrate anticoagulation in continuous renal replacement therapy: is metabolic fear the enemy of logic? A systematic review and meta-analysis of randomised controlled trials. Life. (2023) 13:1198. doi: 10.3390/life13051198 37240843 PMC10221969

[31] Zhang W Bai M Yu Y Li L Zhao L Sun S . Safety and efficacy of regional citrate anticoagulation for continuous renal replacement therapy in liver failure patients: a systematic review and meta-analysis. Crit Care. (2019) 23:22. doi: 10.1186/s13054-019-2317-9 30678706 PMC6345001

